# Differential contribution of cis and trans gene transcription regulatory mechanisms in amygdala and prefrontal cortex and modulation by social stress

**DOI:** 10.1038/s41598-018-24544-3

**Published:** 2018-04-20

**Authors:** Eli Reuveni, Dmitry Getselter, Oded Oron, Evan Elliott

**Affiliations:** Bar Ilan University Faculty of Medicine, Hanrietta Sold 8, Safed, 13215 Israel

## Abstract

While both individual transcription factors and *cis*-acting sites have been studied in relation to psychiatric disorders, there is little knowledge of the relative contribution of *trans*-acting and *cis*-acting factors to gene transcription in the brain. Using an RNA-seq approach in mice bred from two evolutionary-distinct mice strains, we determined the contribution of *cis* and *trans* factors to gene expression in the prefrontal cortex and amygdala, two regions of the brain relevant to the stress response, and the contribution of *cis* and *trans* factors in the prefrontal cortex after Chronic Social Defeat (CSD) in mice. More genes were regulated by *cis*-regulatory factors in both brain regions, underlying the importance of *cis*-acting gene regulation in the brain. However, there was an increase in genes regulated by *trans*-regulatory mechanisms in the amygdala, compared to the prefrontal cortex. These genes were involved in synaptic functions, and were enriched for binding sites for transcription factors, including Egr1. CSD induced an increase in genes regulated by *trans*-regulatory mechanisms in the prefrontal cortex, and induced a pattern similar to the unstressed amygdala. Overall, we show brain site-specific patterns in *cis* and *trans* regulatory mechanisms, and show that these patterns can be modified by a psychological trigger.

## Introduction

A correct understanding of gene expression regulatory mechanisms in the brain is essential to understanding the relationship between genotype and phenotype. Both *cis*-acting and *trans*-acting factors have the potential to regulate gene expression patterns in the brain and to mediate the interaction between environment and gene expression. *Cis*-acting factors are mechanisms that affect gene expression only on the same chromosomal allele, while *trans*-factors act equally on both alleles. Transcription factors and long noncoding RNAs are a classic example of *trans*-acting factors. *Cis*-acting factors generally include regulatory genomic regions, such as enhancers, as well as epigenetic marks. Many studies in gene expression regulation in neuropsychiatry have focused on *trans*-acting mechanisms, mainly the roles of gene transcription factors, such as cAMP response element-binding protein (CREB)^1^ and Fosb^2^, which were shown to mediate behavioral effects of stress. However, *cis*-regulatory mechanisms are known to play specific roles in behavioral susceptibility to stress, and have been receiving increased interest in the past decade. For example, *cis*-regulatory regions of FKBP mediates the behavioral susceptibility to childhood trauma through regulation of three dimensional chromatin structure^3^. Nonetheless, there is little knowledge of the relative contribution of *cis* and *trans* regulatory pathways in gene expression in the brain at the whole genome level. This knowledge is necessary to direct future research in psychiatric genetics towards the most relevant regulatory mechanisms.

Recent RNA-seq based methods have been developed to more fully determine the contribution of *cis* and *trans*-acting factors to gene expression. By comparing the gene expression differences between two genetically distinct organisms to the gene expression difference among the alleles in the F1 offspring, we can differentiate between genes whose expression differences are due to *cis*-regulatory mechanisms or *trans*-regulatory mechanisms. Those genes that show gene expression differences in the different alleles of the F1 hybrids that are comparable to the gene expression differences among the individual organisms are considered to be regulated by *cis*-acting factors.

These methods have been applied to probe the evolution of gene expression, and the possible roles of these two factors in complex traits and disorders. Originally, this method was used to probe the contribution of *cis* and *trans* regulation to gene expression divergence among different species of drosophila^4^, where they found that *trans*-regulatory changes were responsible for a large percentage of gene expression variation among species. In addition, this method was used in the genetically distant mouse models, C57/Bl and CAST/EiJ, to determine that *cis* regulation plays a particularly important role in the evolution of gene expression in the mouse liver^5^. However, there has been little exploration of the role of *cis* vs. *trans* factors in allele-specific gene expression in the brain, and particularly in regions important for cognition or psychopathologies. Of particular interest, there has been little exploration of how these regulatory mechanisms may be affected by physiological or psychological stressors. However, a recent work in four different peripheral tissues has found that allele-specific gene expression can be partly explained by environmental factors, and not only genetic factors^6^. Therefore, it is plausible that allele-specific gene expression in the brain may be effected by environmental factors, such as psychological stress.

The amygdala and prefrontal cortex play primary roles in the psychological response to stress^7^. Amygdala is a primary region in the limbic brain that is responsible for anxiety and fear-based behaviors. Prefrontal cortex sends excitatory signals to the amygdala, which activate GABAergic interneurons. Therefore, the prefrontal cortex inhibits amygdalar activity, and helps to attenuate the stress response. Of interest, decreased activity in the prefrontal cortex has been correlated with post-traumatic stress disorder^8^.

In this study, our first aim is to determine the contribution of *cis* and *trans* regulatory mechanism to gene regulation in the amygdala and prefrontal cortex, with a particular interest in possible brain area specificity. The second aim is to understand how chronic social defeat (CSD) stress, a psychological stressor, may change the equilibrium between trans and *cis* regulatory mechanisms in the brain. As such, this will help us to understand stress-induced global changes in gene regulatory mechanisms in the brain, and the contribution of *cis* and *trans* regulatory mechanisms to overall gene expression.

## Results

### Gene regulatory mechanisms in the prefrontal cortex and amygdala

In order to determine the contribution of *cis*-acting and *trans*-acting regulatory pathways on gene expression divergence in the brain, we utilized the C57Bl and CAST/EiJ mice models, which exhibit a very high rate of single-nucleotide polymorphism (SNP) divergence between the species. We generated F1 hybrids of C57Bl and CAST/EiJ, as well as the reciprocal cross. Subsequently, half of the mice were subjected to CSD. In short, this involves the placement of the mouse with a bully mouse for five minutes, followed by housing with the bully mouse with a divider between them. This stress is then repeated for 10 days, with a different bully mouse each day. Fourteen days after the end of the stress protocol, RNA was extracted from the prefrontal cortex. Unstressed mice were housed during the same period with two mice per cage, with a divider between them, followed by RNA extraction from both the prefrontal cortex and amygdala. These experiments were carried out with the F0 strains (C57/Bl and CAST/EiJ respectively), as well as with the F1 hybrids.

**Figure 1:**
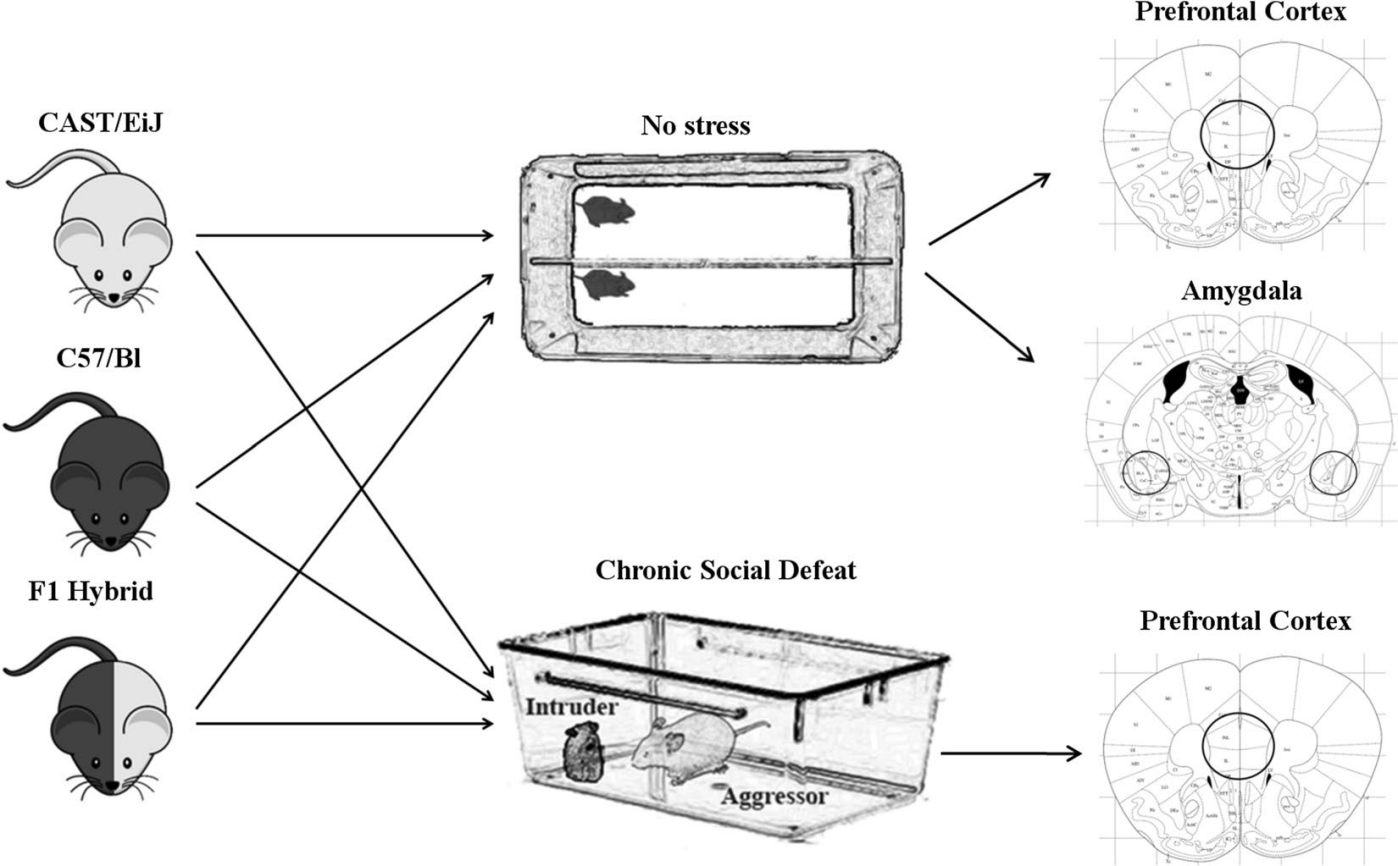
Experimental plan. This experiment includes RNA from 18 mice of each of the following genotypes: C57BL/6, CAST/EiJ, and F1 hybrid mice (C57BL/6/CAST/EiJ). From each genotype, half of the mice (9) were kept in an unstressful condition, while the other half was subjected to chronic social defeat. Two weeks after stress, RNA was extracted from the prefrontal cortex and amygdala of the unstressed group (as shown in mouse brain atlas pictures modified from Paxinos and Franklin brain atlas)^27^, and the prefrontal cortex of the chronic social defeat group. Three mice were group were pooled to one sequencing library. Therefore, there are three sequencing libraries for every group (prefrontal cortex-C57BL/6-unstressed, amygdala-C57/BL/6-unstressed, etc.), for a total of 27 libraries.

For each experimental group, three sequencing libraries were prepared, with each library containing a pool of RNA from three separate mice, followed by 100 base-pair single end sequencing. Sequences from F0 mice (pure C57/Bl or CAST/EiJ) were mapped to their respective transcriptome, while libraries from F1 hybrids were mapped to a hybrid transcriptome reference including C57 and CAST alleles (see materials and methods). *Cis*-*Trans* analysis was carried out for each of the following experimental groups: unstressed amygdala, unstressed PFC, and chronic social defeat PFC (Fig. 1, Materials & Methods).

**Figure 2:**
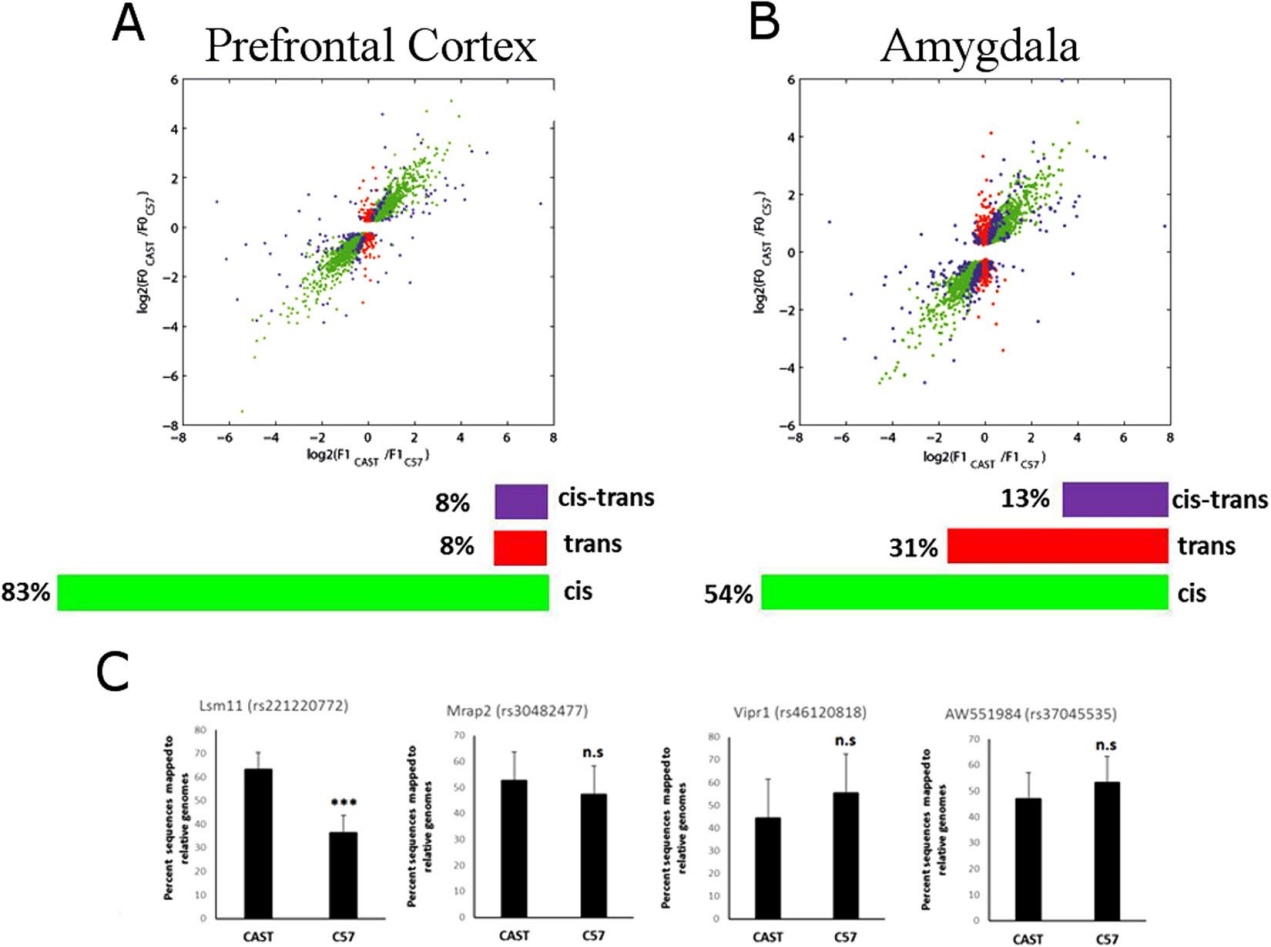
*Cis* and *trans* regulatory mechanisms in the prefrontal cortex and amygdala. Each plot summarizes the allele-specific gene expression analysis of RNA-seq data from the prefrontal cortex (**A**) and amygdala (**B**). Each dot represents a gene, and each is color coded according to the its form of regulation. (**C**) Reduced Representative high throughput sequencing was carried out on F1 amygdalar samples to validate the RNA-seq results of four specific genes. One cis regulated gene, Lsm11, and three trans regulated genes, Mrap2, Vipr1, and AW551984 were studied. Reduced Representative sequencing confirms an allele-specific transcription of Lsm11, verifying cis-regulation, and no allele-specific transcription of Mrap2, Vipr1, and AW551984, verifying their trans-regulation. ***p < 0.001 n.s = not significant. two tailed t-test. Error bars represent standard deviation.

Our first aim was to determine the contribution of *cis* and *trans* regulatory mechanisms in brain transcription in unstressed conditions, and to understand if there are any brain-site specific differences in gene regulation. In both the prefrontal cortex and the amygdala, 20% of genes displayed a significant divergence in expression between C57/Bl and CAST/EiJ, allowing for closer examination of the regulatory mechanisms of those genes. This coincides with a recent study that determined a 23% divergence of gene expression in the embryo. In the prefrontal cortex, 84% of divergent genes were regulated only *in cis*, while only 8% of genes were regulated only *in trans* (Fig. 2A). An additional 8% of genes were regulated by both *cis* and *trans* mechanisms (*cis*-*trans*). However, in the amygdala, we deciphered a different gene regulatory pattern, where only 55% of genes were regulated *in cis*, and 32% of genes were regulated *in trans*, which is a four-fold increase compared to the prefrontal cortex (Fig. 2B). Therefore, *in trans* regulatory mechanisms are much more common in the amygdala than in the prefrontal cortex.

In order to verify the statistical pipeline and our RNA-seq results, we performed gene-specific targeted next generation sequencing on the F1 amygdalar samples of four genes to decipher if they show the same cis/trans orientation as determined in the whole transcriptome RNA-seq analysis. In fact, we found that the gene lsm11 displayed allele-specific gene expression in the F1 samples, validating that this gene is regulated *in cis*, while Mrap2, Vipr1, and AW551984 displayed no allele-specific expression, validating that they are regulated *in trans* (Fig. 2C, Table S9). Therefore, we were able to validate our RNA-seq pipeline in a small subset of genes.

**Figure 3:**
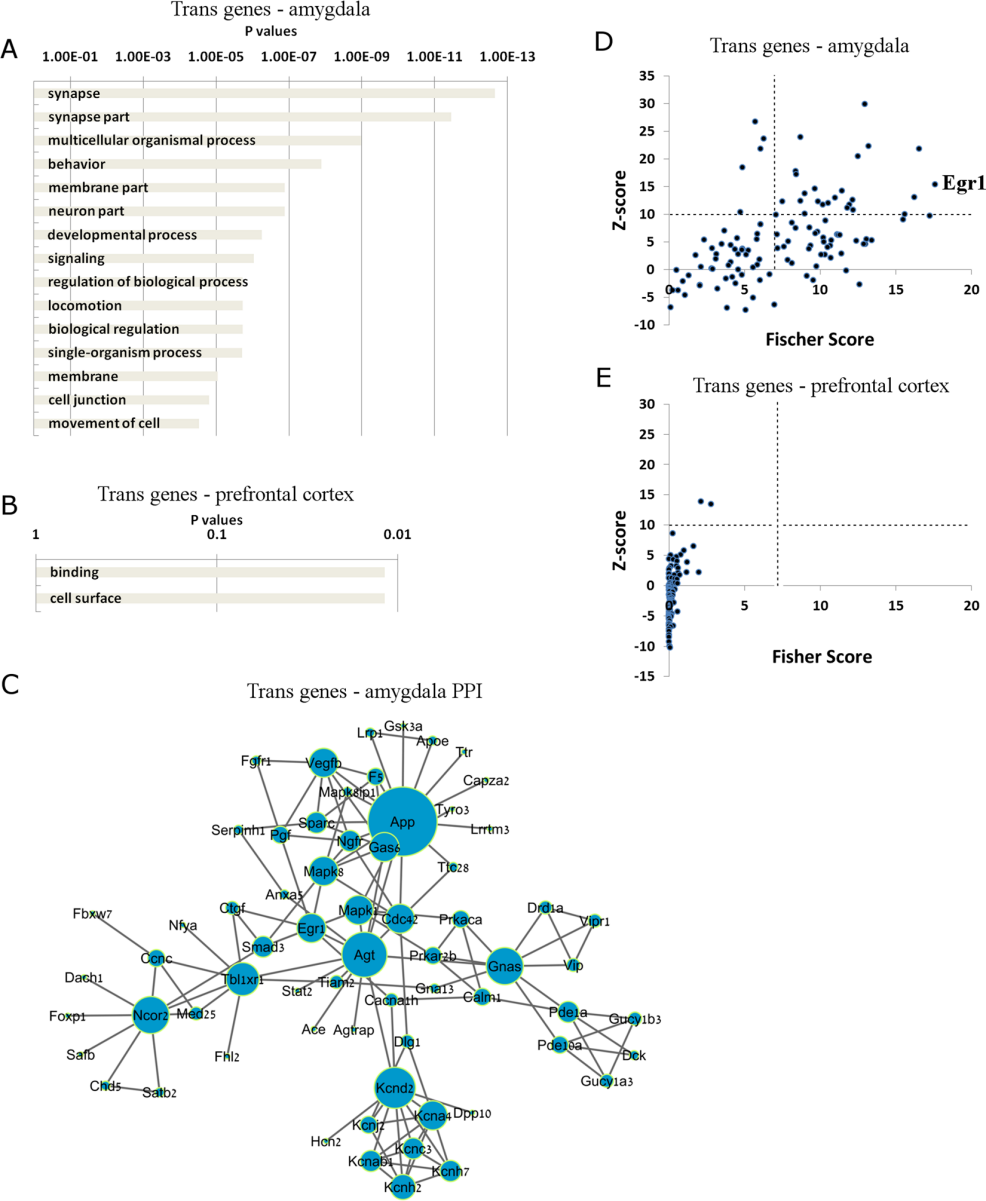
Genes regulated *in trans* specifically in amygdala are involved in synaptic function and enriched for many transcription factor sites. (**A**,**B**) Gene ontology pathways of genes that were regulated by *trans* mechanisms specifically in the amygdala (**A**) and in the prefrontal cortex (**B**). (**C**) PPI analysis of the genes that are regulated *in trans* specifically in the amygdala. (**D**,**E**) Transcription Factor binding site enrichment analysis using the Opposum tool in genes that were regulated *in trans* specifically in the amygdala (**D**) and in the prefrontal cortex (**E**). According to previously published data, we set a cutoff for significantly enriched sites at Z-score greater than 10 and Fisher score greater than 7.

To determine if genes responsible for specific biological processes are effected by *in trans* regulatory mechanisms specifically in the amygdala, gene ontology analysis was performed. Genes regulated *in trans* in the amygdala were enriched for nearly thirty biological pathways, including synapse, synapse part, and behavior (Fig. 3A). Only two pathways were enriched in genes that were regulated *in trans* specifically in the prefrontal cortex, which were binding and cell surface (Fig. 3B). These results indicate that genes involved in neuronal and behavioral functions are more likely to be regulated *in trans* in the amygdala, compared to the prefrontal cortex.

In order to gain more insight into the biological function of genes that were regulated *in trans* specifically in the amygdala, we performed protein-protein interaction analysis (PPI). We determined a large cluster of interacting proteins, which include Kcnd2, Agt, App as central hub proteins (Fig. 3C). Gene ontology analysis of this cluster determined the enrichment of proteins involved in *cation channel complex* and *voltage gated potassium channel complex*. The PPI analysis strengthens the findings that genes involved in neuronal function, such as neuronal activity, are regulated *in trans* specifically in the amygdala

Since *trans* regulatory mechanisms include transcription factors, we considered that particular gene transcription binding sites may be enriched amongst the genes that are regulated *in trans* specifically in the amygdala. Using Opossum tool, we tested for transcription factor site enrichment in our sets of genes. We find that transcription factor binding sites upstream of 23 *trans* expressed genes in the amygdala were likely to be enriched in common transcription factors (Fig. 3D, Table S1). Of particular interest, the transcription factor site with the highest fisher score was Egr1. Egr1 is a transcription factor that is critical in neuronal activity and synaptic plasticity^9^. Of equal importance, Egr1 is a glucocorticoid-responsive factor that mediates the transcriptional response to psychological stress^10,11^. In contrast, no transcription factor sites were enriched in the gene set that is regulated *in trans* specifically in the prefrontal cortex (Fig. 3E, Table S2). In summary, the enrichment of neuronal and synaptic genes that are regulated *in trans* in the amygdala may be partially explained by differential regulation of genes that contain the Egr1 transcription factor site. These results highlight brain-site specific differences in gene expression regulatory mechanisms.

### Effect of stress on gene regulatory patterns in the PFC

**Figure 4:**
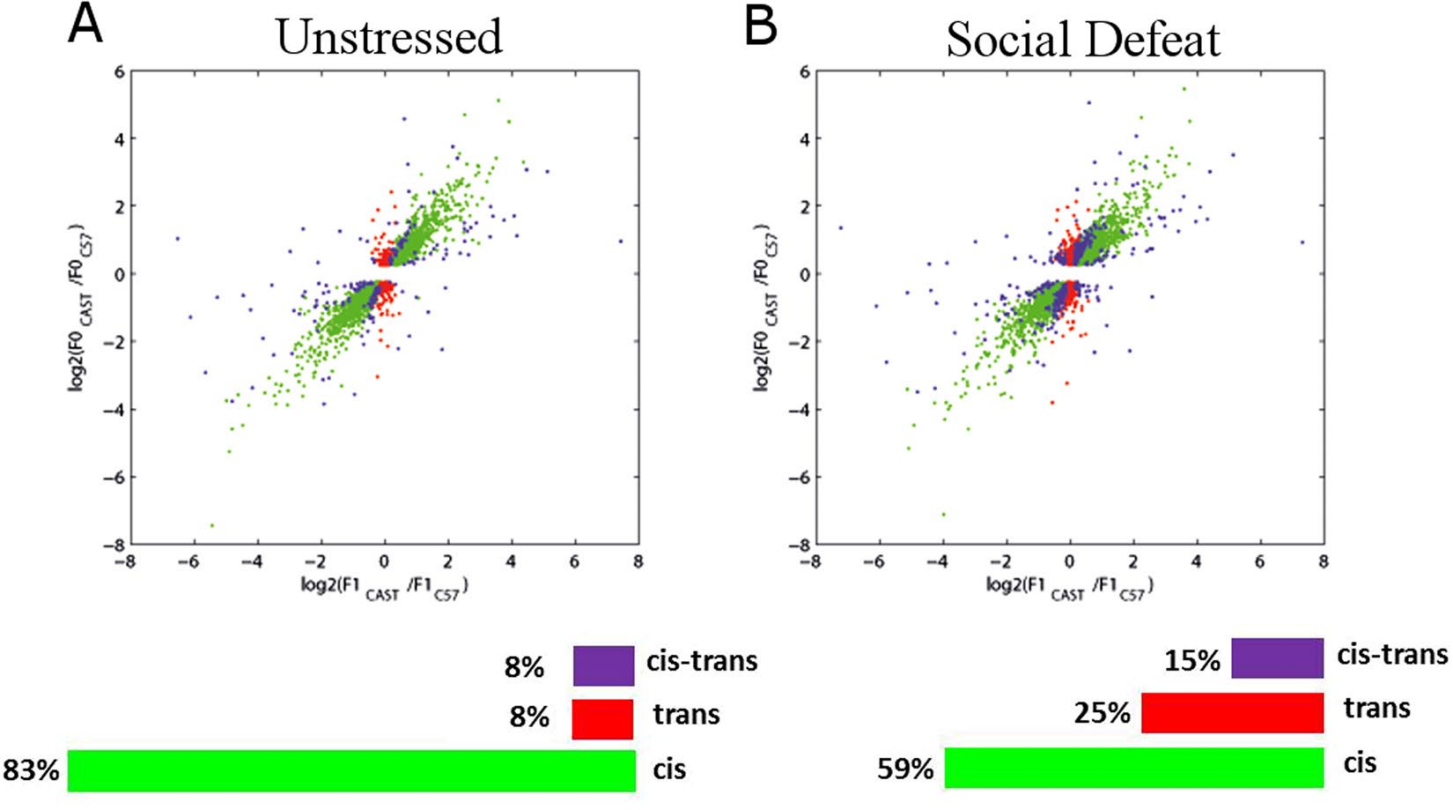
*Cis* and *trans* regulatory mechanisms in the unstressed and stressed prefrontal cortex. Each plot summarizes the allele-specific gene expression in the unstressed (**A**) and stressed (**B**) prefrontal cortex. Each dot represents a gene, and each is color coded according to the its form of regulation.

In order to determine if the balance between *cis* and *trans* regulatory mechanisms in the brain is amendable to psychological stress, we compared the *cis* and *trans* regulation levels in the prefrontal cortex between control and stressed mice. We focused on the prefrontal cortex because a plethora of studies have determined long term changes in functionality and synaptic morphology in this brain region in response to stress, which is correlated to dysregulated stress response and Post-traumatic stress disorder. In stressed animals, 25% of the genes were regulated *in trans*, compared to 8% in the nonstressed animals (Fig. 4A,B). *Cis*-*trans* regulated genes also went up from 8% to 15%, while *in cis* only genes decreased from 83% to 59%. These results indicate that CSD induces an increase in *in trans* regulatory mechanisms in the prefrontal cortex.

**Figure 5:**
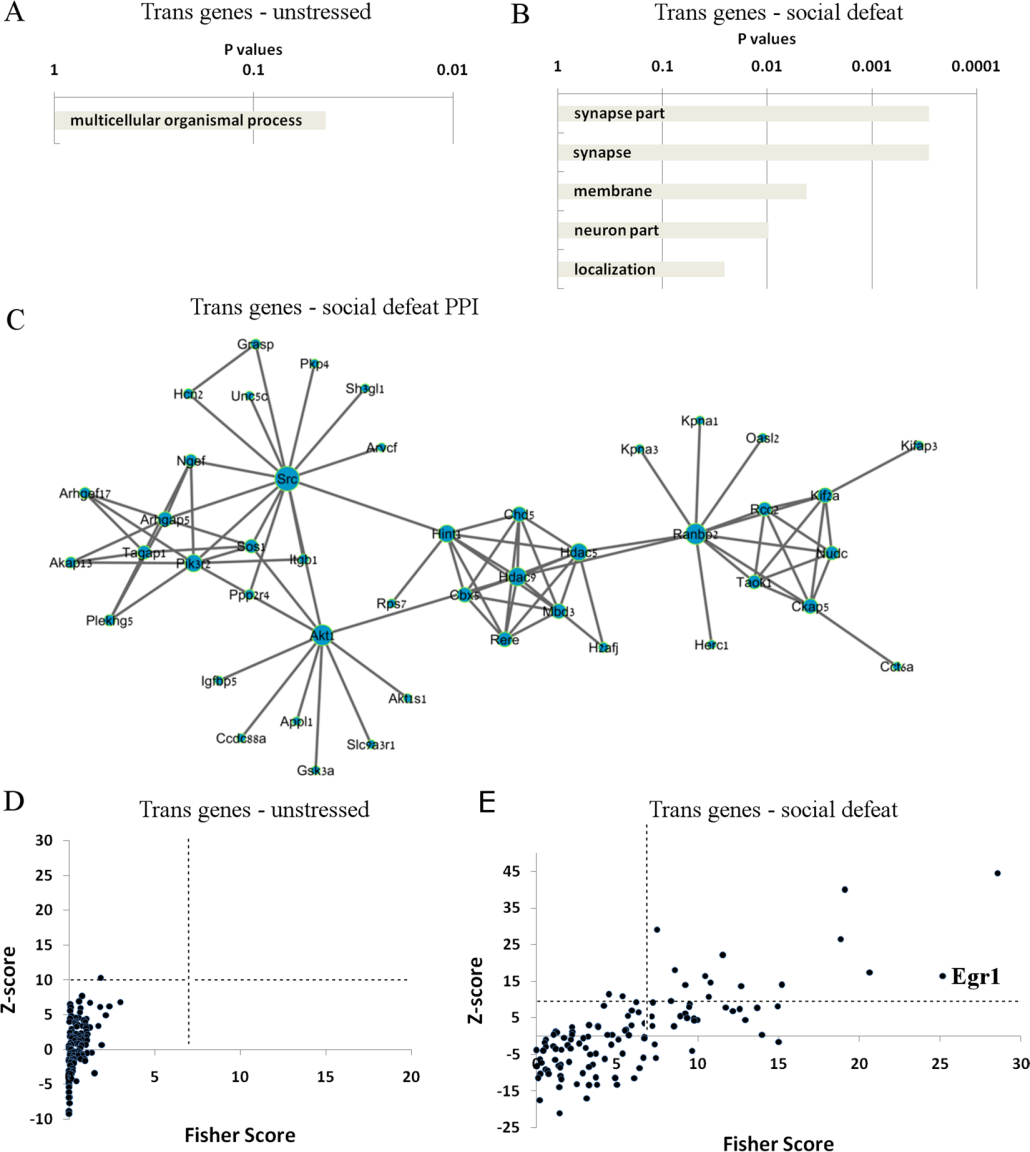
Social Defeat Stress induces changes in population of *trans*-regulated genes in the prefrontal cortex. Gene ontology pathways of genes that were regulated by *trans* mechanisms specifically in prefrontal cortex of unstressed mice (**A**) and in mice subjected to chronic social defeat (**B**). (**C**) PPI analysis of the genes that are regulated *in trans* specifically after social defeat in the prefrontal cortex. (**D**,**E**) Transcription Factor binding site enrichment analysis using the Opposum tool in genes that were regulated *in trans* specifically in unstressed mice (**D**) and in mice subjected to chronic social defeat (**E**). According to previously published data, we set a cutoff for significantly enriched sites at Z-score greater than 10 and Fisher score greater than 7.

Genes that were regulated *in trans* only in the unstressed group fell into two gene ontology groups, organismal developmental processes and developmental processes (Fig. 5A). In contrast, genes that were regulated *in trans* only in the stressed group fell into four gene ontology groups, including synapse, membrane, and neuron(Fig. 5B). Of interest, these are some of the same categories that were regulated *in trans* in the unstressed amgydala. In addition, PPI analysis of the *in trans* genes in the stressed group revealed a cluster of interacting proteins with Akt1 and Src, two important signal transduction factors, as central hubs of the cluster (Fig. 5C). Akt1, in particular, is a central factor in signal transduction in neurons.

Using opossum tool, we found 13 transcription factor binding sites that were enriched among the genes that are regulated *in trans* only in the defeat group, while there were no transcription factor sites among the unstressed group (Fig. 5D,E, Tables S3, S4). Again, Egr1 was one of the top transcription factors enriched in the defeat group. Overall, the data suggests a shift in *cis*-*trans* regulation in the stressed prefrontal cortex towards a higher abundance of *trans* regulation, particularly in genes involved in neuronal and synaptic activity, as well as in genes with the stress-responsive element Egr1 transcription factor site.

The differential enrichment of Egr1-element containing genes among different experimental conditions could be due to multiple mechanisms, including epigenetic modifications at the Egr1-binding site, differential recruitment of Egr1 to its target site, or differential expression of Egr1. We performed real time PCR for Egr1 levels in all experimental groups to understand if differential gene expression of Egr1 could explain our findings. In both CAST and C57 mice, Egr1 was expressed more in the prefrontal cortex than in the amgydala (Supplementary Figure S1). This is surprising, considering that Egr-1-element containing genes were regulated *intrans* specifically in the amygdala. In addition, there was no difference in Egr1 levels between unstressed and CSD prefrontal cortex. Therefore, the enrichment of genes with Egr1 element in trans-regulation does not correlate with increase in Egr1 levels, suggesting that a different epigenetic mechanism is more likely to explain our findings.

## Discussion

In our study, we determine that the contribution of *cis* and *trans* regulatory mechanisms to gene expression is brain-site specific, and can be modified by a psychological stress. One of the first novel findings from this study is the high amount of *cis* regulation in gene expression in the brain. Most studies of gene transcription in the brain have focused on the roles of trans regulatory elements, such as transcription factors, microRNAs, etc. However, our results indicate that *cis-*only regulatory mechanisms are a prominent gene regulatory factor in the mouse brain, and are therefore relevant targets for studies into the biology of mental conditions. A few previous studies have discovered SNPs in *cis*-regulatory regions that are responsible for differential expression of FKBP5^3^ and BDNF^12^, and that correlate with susceptibility for post traumatic stress disorder and clinical depression. Recent studies have also identified *cis* elements that are essential for proper forebrain development^13,14^. However, *cis* elements were not studied extensively at the whole-genome level in the past, mainly due to the technical effort involved. Only recently have these technical advances been available to decipher the roles of *cis*-elements in biological processes and human disease^15^. As methods to interrogate *cis* factors improve, more studies will focus on the contribution of *cis*-acting factors in gene transcription.

A previous study used eQTL analysis of gene expression from human twins to determine that environmental influences may contribute to *cis* and *trans* regulatory mechanisms^16^. Our data significantly adds to this area of study by providing direct evidence that a particular type of environmental influence, chronic social defeat, may influence the contribution of *cis* and *trans* regulatory factors in the brain. In the prefrontal cortex, stress induced a shift towards regulation by *trans* mechanisms. This suggests that *cis* mechanisms are more involved in basal gene transcription levels, while *trans* mechanisms, perhaps transcription factors, play a more relevant role in environment-induced gene transcription changes, at least in the PFC. This idea is supported by recent research showing that *cis* regulatory elements are particularly important for forebrain development^13,14^. Therefore, we may speculate that *cis* regulatory elements play a primary role in the development and establishment of basal gene regulation patterns, while trans regulatory mechanisms have been developed partially to regulate gene expression in response to environmental factors.

One of the highly enriched transcription factor sites in the *trans*-regulated genes in both the control amygdala and stressed prefrontal cortex groups was the Egr1 site. Egr1 is a neuronal activation marker that has been shown to be involved in stress pathways. For example, Egr1 activation by serotonin induces the transcription of glucocorticoid receptor^11^. Early life stress can inhibit the binding of Egr1 to the glucocorticoid receptor gene specifically in the hippocampus through methylation of the Egr1 binding site. However, early life stress also increases the expression of Egr1 in the hippocampus, suggesting that Egr1 activity at other sites may increase^17^. A separate study found that inhibition of Egr1 specifically in the prefrontal cortex induced social anxiety behaviors^18^. Therefore, Egr1 activity in the prefrontal cortex is relevant to the social stress used in our study. It is not quite clear the exact mechanism by which genes with Egr1 site are enriched in tran-regulated genes in the amygdala. Egr1 is not expressed more in the amygdala than in the PFC. However, we may speculate that signal transduction mechanisms that are involved in Egr1 activation, or that direct Egr1 to the chromatin, may be involved in increased Egr1-related *trans* regulation in the amygdala.

To understand more fully the tissue-specificity of *trans* and *cis* regulatory effects, we can compare our findings in the brain to previously published findings of these mechanisms in the liver. A previous study by Goncalves *et al*. used the same experimental design of CAST/EiJ and C57/Bl hybrid breedings to decipher these mechanisms in the liver^5^. In their study, approximately half of the genes that showed expression divergence were regulated by both *cis* and *trans* mechanisms (*cis*-*trans*). In our study, this was a relatively rare event. First, this difference seems to indicate that genes in the brain are much less likely to be effected by both regulatory mechanisms, but are only effected by either *cis* or *trans*. Second, this also shows that a much higher percentage of genes in the liver are regulated, at least partially, by *trans* mechanisms, compared to the brain. Again, this difference indicates that *cis* regulatory mechanisms, such as enhancer elements and epigenetic marks, may play a more central role in gene regulation in the brain, compared to the liver.

One limitation of this study, and many previous studies, is that we rely on two specific mice strains, CAST/EiJ and C57/Bl, where 80% of genes were conserved in the brain between the two strains. Therefore, these genes could not be probed for effects of *cis* and *trans* regulatory mechanisms. It is highly likely that the use of any other two mouse strains would also give at least 80% conserved genes, since CAST/EiJ and C57 are known to display significantly different gene expression in multiple studies. However, one possible method to increase the amount of nonconserved genes would be to increase the amount of mouse models used to three or four, and to probe all genes that are unconserved in at least two of those models. This method could be applied to future studies.

It has previously been established that mice can display a very variable behavioral response to CSD. Some mice display social avoidance after this stressor, and are therefore referred to as “susceptible” to CSD, while other mice do not display social avoidance, and are therefore referred to as “resilient^19,20^. Previous studies have shown that differential epigenetic marks in the brain correlate with the phenotype for susceptibility^1^. In our study, we tested the effects of CSD on gene regulation, but did not test how these effects may correlate with susceptibility to CSD-induced social avoidance. As such, we don’t know if the stress-induced shift to trans regulatory mechanisms occurs equally in both susceptible and resilient mice, or if this shift may be more pronounced in one of the behavioral subgroups. Therefore, this is an additional limitation of our study.

Overall, the current study widens our understanding of genetic regulation in the brain, and underlies its deep complexity, both in terms of spatial regulation (brain site) and how is it amendable by environmental factors. Moreover, these findings emphasize the importance of looking at cis-regulatory elements in psychiatric genetics.

## Materials and Methods

### Animals

Mice were given food and water *ad libitum*. Adult C57BL/6 and CAST/EiJ male mice were maintained, and hybrid C57BL/6/CAST/EiJ mice were produced by the breeding of C57BL/6 male mice and CAST/EiJ female mice, and vice versa. All experimental procedures were carried out in accordance with the experimental protocol approved by the animal care and use committee of Bar Ilan University under protocol number 20-5-2012.

### Social Defeat Protocol

The social defeat protocol was carried out as previously described^19^ on all three mice groups: C57BL/6, CAST/EiJ, and hybrid C57/BL/6/CAST/EiJ. The test mouse was placed in the home cage with an aggressive ICR retired breeder mouse, and they interacted physically for five minutes. ICR (CD-1) mice are an outbred mouse strain which displays high aggressiveness, particularly after repetitive breeding. It is commonly used as an aggressor mouse in the social defeat paradigm^19^. During this time, the ICR mouse attacked the intruder mouse. A perforated clear plexiglass divider was then placed between the animals and they remained in the same cage for 24 hours to allow sensory contact. The procedure was then repeated with an unfamiliar ICR mouse for each of the next ten days. Unstressed mice were housed two mice per cage, with a divider between them.

### RNA extraction and library construction

Two weeks after the end of the social defeat stress, mice were sacrificed by rapid decapitation and brains were removed. The PFC was isolated using a 13 gauge needle, and was frozen on dry ice. The amygdala was isolated using a 16 gauge needle. Total RNA was extracted using RNAeasy Mini Kit (Qiagen). Sequencing libraries were prepared using Illumina Truseq RNA Sample Preparation kit. Three different libraries were produced from each experimental group, with each library being produced from RNA of a pool of three different mice. Therefore, each experimental group contains nine mice. Sequencing was performed on the Hiseq 2500 machine in the Bar Ilan University Faculty of Medicine Genome Center.

### Mapping and allele specific expression analysis

Sequence reads from F0 and F1 mice strains were aligned to transcriptome reference using Bowtie2^21^. For F0 we have used CAST and C57 transcriptome reference for each strain reads while reads from F1 strains were aligned to a hybrid reference transcriptome containing both reference origins. Both mouse transcriptomes are based on Wellcome Trust Mouse Genomes Project Release^22^.

In order to calculate gene expression level for F0 and allele specific expression for the F1 reads we have used MMSEQ^23^ which uses a Bayesian approach to count reads to alleles even in the presence of low number of mutations. After obtaining read count for each gene we have normalized read count according to the method described in DEseq^24^. In order to exclude non-expressed genes we have considered gene as expressed if at least in one of the F0 or F1 strains contained read count >10 reads. Our filtering procedure allowed us to detect 13,274 genes for the *cis*-*trans* analysis.

### *Cis/trans* analysis

Our experimental of F0 and F1 mice allowed us to assess *cis* and *trans* regulation. Briefly, gene expression ratios in F0 and F1 can be used to find imbalance that may attribute to the mode of regulation. We assume that genes which are expressed equivalently in F1 between CAST and C57, but differ in F0 are suspected as *trans* regulated genes, and genes that differentially express in F0 and in F1 are under *cis* regulation. We have used the method described in McManus *et al*.^4^ to determine for *cis* or *trans* regulation.

In short, the F0 and F1 data sets were analyzed for differential expression using the binomial exact test, followed by FDR analysis, which produced a list of differentially expressed genes (FDR < 0.01) in these two data sets (Table S5). Differential gene expression in F0 is evidence of expression divergence, and differential expression in F1 is evidence of *cis*-regulatory divergence. To test for *trans*-regulatory divergence, fisher’s exact test was used to compare species-specific mRNA abundance ratios between the F0 and F1 samples. If the ratios of mRNAs were significantly different between the F0 and F1 data sets (FDR < 0.01), then there was a *trans*-regulatory effect. This analysis was used to produce lists of genes regulated by *cis*, *trans*, or *cis* and *trans* (*cis*-*trans*) regulatory mechanisms in each of the three experimental groups (amygdala, prefrontal cortex, stressed prefrontal cortex) (Tables S6, S7, S8).

### Gene ontology analysis and transcription factor site enrichment analysis

Enrichment analysis was done using Ontologizer^25^ to look for gene enrichment regulated *in trans* in the following gene sets: (1) unique gene set from control amygdala and control PFC after excluding intersect genes, (2) unique gene sets from control PFC and defeat PFC after excluding intersect genes. Enriched GO group allowed us later on to determine whether *trans* activated genes in control amygdala differs from defeat PFC in order to assess related patterns if functionality between amygdala and prefrontal cortex under different stress conditions.

The Opposum online tool^26^ was used to identify transcription factor sites that are enriched in our data sets. In short, the Opposum tool aligns the genes of interest to the JASPAR database of known transcription factor binding sites. Then two statistical tools are used to determine enrichment of specific sites in our genes of interest. These statistical tests include a simple binomial distribution test, resulting in a Z-score, and a fisher’s exact test. Transcription factor binding sites that meet the default threshold of statistical significance in both tests (Z-score greater than 10 and fischer score greater than 7) are considered significantly enriched in the dataset.

### PPI network analysis

Protein-Protein Interactions were produced with the STRING database, which predicts association between proteins according to several types of evidence. In this analysis, the prediction evidence used were based on neighboring genes (Neighborhood), co-expressed genes (Co-expression), experimental data (Experiments), additional curated databases (Databases) and text mining data (Text Mining). STRING then calculates a combined confidence score of possible association between proteins based on the evidence. In this analysis, the confidence score was set to the high threshold of 0.7 to generate the most likely interactions. To visualize the network, we imported the combined confidence score from the STRING analysis and loaded it onto Cytoscape(version 3.2). Network topology was calculated with the edge-weighted forced directed layout, using the combined confidence score imported from STRING. To identify subPPI networks, the MCODE algorithm of the Clustermaker v2 application for Cytoscape was used under default attributes and the Fluff cluster finding option.

### Real Time PCR

cDNA was synthesized from RNA samples using the High Capacity cDNA Reverse Transcriptase kit (Applied Biosystems). Real time PCR was performed using FastStart Universal SYBR Green Master (Rox) (Roche) and analyzed with ViiA™7 Real-Time PCR System (Applied biosystems). Technical triplicates were performed on each biological sample. Primers used are- HPRT: Forward:GCAGTACAGCCCCAAAATGG Reverse:GGTCCTTTTCACCAGCAAGCT. Egr1:Forward: TATACTGGCCGCTTCTCCCT.

Reverse: AGAGGTCGGAGGATTGGTCA.

### Targeted Next Generation Sequencing

Targeted sequencing was used to validate allele-specific expression in four genes in the F1 amygdalar samples. Primers were designed to amplify products containing CAST/C57 specific SNPs (Table S10). cDNA samples were submitted to the Genome Center at “Barts and The London School of Medicine and Dentistry” (London, UK). Briefly, cDNAs were PCR amplified using the FastStart™ High Fidelity PCR System by Roche (4738292001, Sigma-Aldrich). The separate barcoded PCR products were then pooled and sequenced on Illumina MiSeq using their 300 cycle v2 kit (MS-102-2002), at 5pM (Illumina, CA, USA). GREP command line was used to count in the fastq files for each of the possible sequences, and percent of each allele-specific sequences are reported in Table S9 and Fig. 2C.

## Electronic supplementary material

Supplementary Figures

Supplementary Tables 1-10
