# Supplementary material for: Differential contribution of cis and trans gene transcription regulatory mechanisms in amygdala and prefrontal cortex and modulation by social stress
Source: Sci Rep. 2018 Apr 20;8:6339. doi: 10.1038/s41598-018-24544-3 (PMC5910421; doi:10.1038/s41598-018-24544-3)
Supplement: Supplementary file 1 — Supplementary Figures [file 41598_2018_24544_MOESM1_ESM.doc]

Differential contribution of cis and trans gene transcription regulatory mechanisms in amygdala and prefrontal cortex and modulation by social stress.

Eli Reuveni^1*^, Dmitry Getselter^1*^,Oded Oron^1^, Evan Elliott^1^

^1^Bar Ilan University Faculty of Medicine

Hanrietta Sold 8 Safed Israel 13215

*The first two authors should be considered join first authors

**Supplementary Information**

**
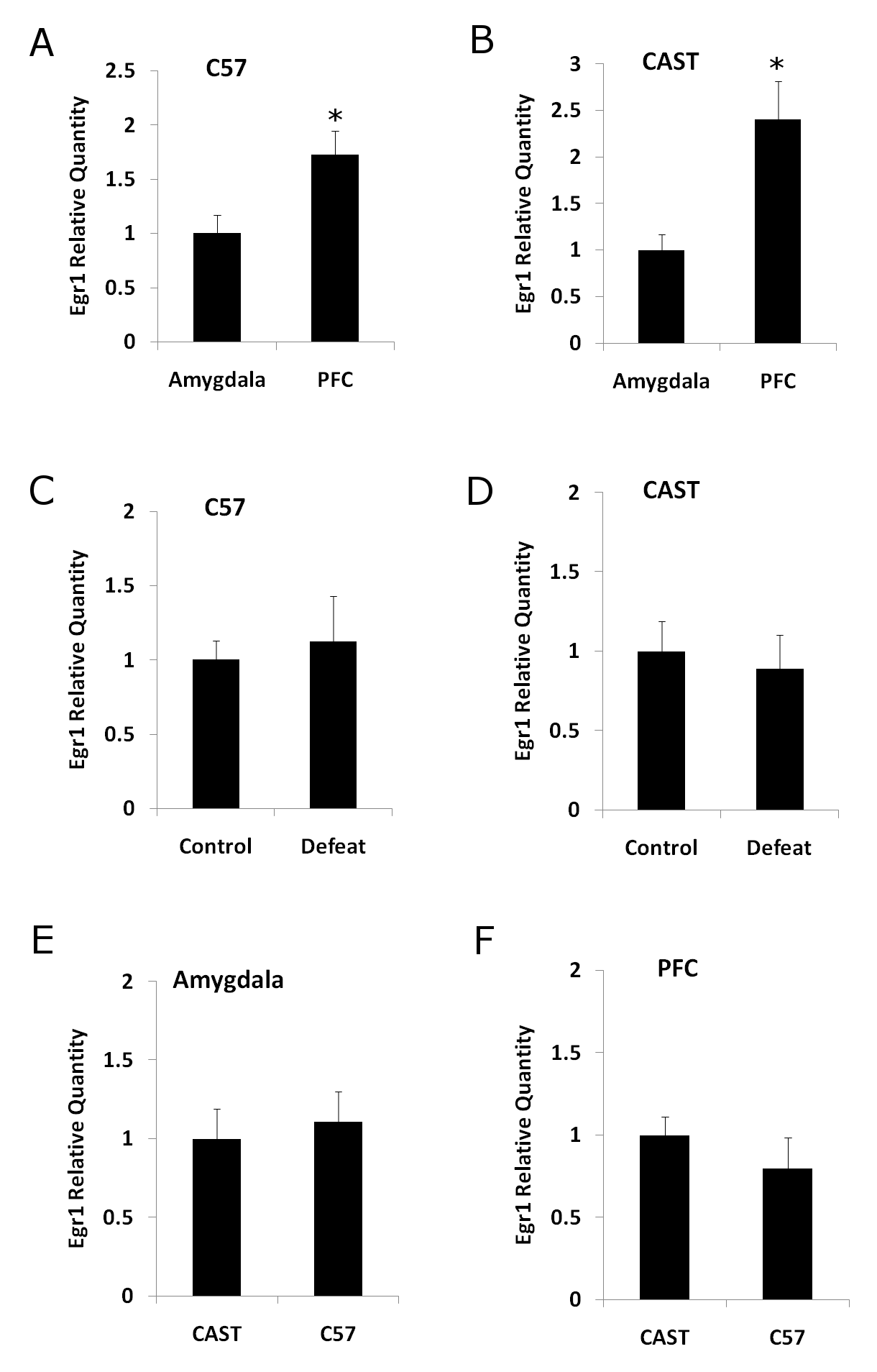
**

**Supplementary Figure 1**

**Brain site-specific and stress-induced Egr1 gene expression**

Real Time PCR analysis of Egr1 gene expression among our different experimental groups. There is significant difference of Egr1 levels between prefrontal cortex and amygdala in both C57 (A) and CAST (B) mice. Social defeat had no effect on gene expression in C57 (C)and CAST (D) mice. There is also no difference in expression of Egr1 between CAST and C57 mice in the amygdala (E) and PFC (F). n=9 in all groups. *=p<0.05 two tailed t-test.

**Supplementary Table Legends**

**S1 Table - Opposum analysis of enriched transcription factor sites in genes regulated *intrans* specifically in amygdala**

**S2 Table - Opposum analysis of enriched transcription factor sites in genes regulated *in trans*specifically in prefrontal cortex**

**S3 Table - Opposum analysis of enriched transcription factor sites in genes regulated *in trans* specifically in unstressed prefrontal cortex**

**S4 Table - Opposum analysis of enriched transcription factor sites in genes regulated *in trans* specifically in stressed prefrontal cortex**

**S5 Table - Lists of genes that are differentially expressed between CAST and C57 in either F0 or F1 hybrids.**

**S6 Table - List of each gene assigned a gene expression regulatory status (*cis*, *trans*, *cis*-*trans*) in the unstressed amygdala, including average normalized read counts for F0 Cast, F0 C57, F1 CAST, and F1 C57.**

**S7 Table - List of each gene assigned a gene expression regulatory status (*cis*, *trans*, *cis*-*trans*) in the unstressed prefrontal cortex, including average normalized read counts for F0 Cast, F0 C57, F1 CAST, and F1 C57.**

**S8 Table - List of each gene assigned a gene expression regulatory status (*cis*, *trans*, *cis*-*trans*) in the stressed prefrontal cortex, including average normalized read counts for F0 Cast, F0 C57, F1 CAST, and F1 C57.**

**S9 Table - Gene specific sequencing analysis in amygdalar samples: Final statistics of F1 reads mapped to specific SNPs from four chosen genes**

**S10 Table - Primers for amplification of specific genes in targeted high throughput sequencing**
